# Shape Analysis of the Patellar Bone Surface and Cutting Plane for Knee Replacement Surgery

**DOI:** 10.1155/2018/6490425

**Published:** 2018-10-24

**Authors:** E. L. Rex, J. Werle, B. C. Burkart, J. R. MacKenzie, K. D. Johnston, C. Anglin

**Affiliations:** ^1^Biomedical Engineering, University of Calgary, Calgary, Canada; ^2^McCaig Institute for Bone and Joint Health, University of Calgary, Calgary, Canada; ^3^Section of Orthopaedic Surgery, University of Calgary, Calgary, Canada; ^4^Dept. of Civil Engineering, University of Calgary, Calgary, Canada

## Abstract

Geometry of the patella (kneecap) remains poorly understood yet is highly relevant to performing the correct patellar cut to reduce pain and to improve function and satisfaction after knee replacement surgery. Although studies routinely refer to “parallel to the anterior surface” and “the patellar horizon,” a quantitative definition of these is lacking and significant variability exists between observers for this irregularly-shaped bone. A 2D-3D shape analysis technique was developed to determine the optimal device configuration for contacting the patellar surface. Axial and sagittal pseudo-X-rays were created from 18 computed tomography (CT) scans of cadaveric knees. Four expert surgeons reviewed three repetitions of the X-rays in randomized order, marking their desired cut plane and their estimate of the anterior surface. These 2D results were related back to the 3D model to create the desired plane. There was considerable variability in perceptions, with intra- and intersurgeon repeatability (standard deviations) ranging from 1.3° to 2.4°. The best configuration of contact points to achieve the desired cutting plane was three pegs centred on the patellar surface, two superior and one inferior, forming a 16 mm equilateral triangle. This configuration achieved predicted cut planes within 1° of the surgeon ranges on all 18 patellae. Implementing this, as was done in a subsequent prototype surgical device, should help improve the success and satisfaction of knee replacement surgery.

## 1. Introduction

**Figure 1:**
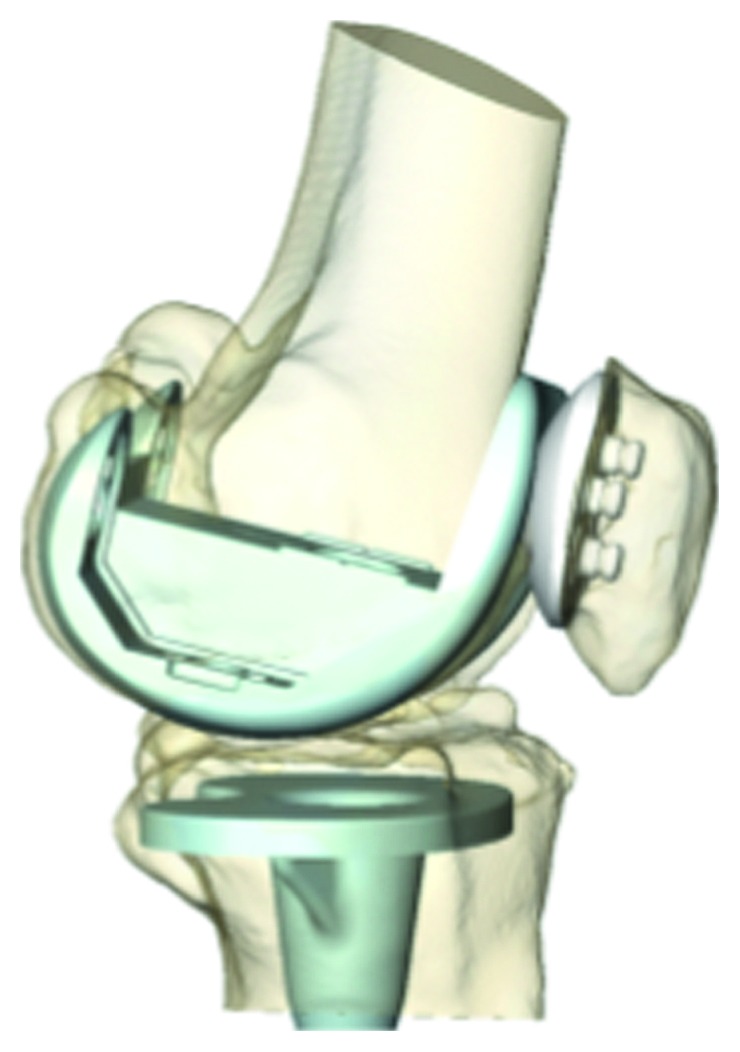
In TKA, the arthritic surfaces of the femur, tibia, and patella are replaced with artificial components to reduce pain and improve function. The patella is normally cut “parallel” to the anterior (front) surface. Holes are then drilled into the flat cut surface to insert the patellar component. An incorrect cut of the patella can result in postoperative pain and complications.

The patella has a critical impact on the outcome of knee replacement surgery (also called total knee arthroplasty or TKA) (Figure 1). An incorrect cut of the patella, meaning too thick or too thin in one or more of the quadrants, can lead to pain [1–3], improper tracking of the patella within the femoral groove [4], and decreased range of motion [5–7], directly affecting the person's activities of daily living and quality of life. Patellar asymmetry occurs in at least 10% of cases, even amongst expert surgeons [8], contributing to the approximately 20% of TKA patients who are not satisfied with the result of their surgery [9, 10].

A variety of devices have been developed to aid surgeons with cutting (resecting) the patella during TKA. Unfortunately, using these devices can still lead to asymmetry and incorrect thickness, resulting in the clinical complications listed above. Computer-assisted surgery (CAS) can be used to improve accuracy [11] but remains invasive due to attaching a marker array onto the bone and is still only as accurate as the placement of the attachment plate. Many surgeons prefer to resect the patella freehand, since they consider the current devices to be inaccurate. A new approach is needed to create a device that is accurate, fast, and noninvasive.

Fundamental to designing a new, noninvasive instrument for patellar resection is determining suitable contact points on the front (anterior) surface. Some devices, such as the standard pliers-like sawguide, which wraps around the patella, have no contact with the anterior surface, relying on the surgeon's judgment to align it with the anterior surface [12]. Others, such as reamers, have a circle of spikes that contact the anterior surface. However, depending on the ridges and hollows on the surface, the plate is sometimes not parallel to the anterior surface, resulting in an asymmetric cut [12, 13]. Symmetry and thickness are also affected when the spikes enter the soft tissue and bone to varying depths [12, 13].

Although various landmark guidelines, such as the medial-lateral (side-to-side) extents, have been suggested for performing the patellar cut, the ultimate goal is to be parallel to the anterior surface, producing a visually rectangular cut, with equal thicknesses in all quadrants [13]. Since asymmetry with respect to the anterior surface, considered to be within ±7° [11], has been correlated with anterior knee pain [2, 3], it is essential to reference a device off of the anterior surface.

Two surprising challenges are to determine what the anterior surface plane is (although the visual goal is a “rectangular” cut, the anterior surface is not flat, and is rarely considered in three dimensions) and to determine whether parallel to this is indeed what looks right to surgeons on the postoperative X-rays. Due to the ellipsoidal and variable shape of the patella, what looks obvious to one person as the anterior surface and the desired resection line is often different from what another person sees [8]. A mathematical definition of the anterior surface plane could be generated, but it was nonetheless essential to confirm that the estimated surface plane corresponds to the resection plane desired by surgeons, thus requiring surgeon input. Statistical shape models have been created of the patellar bone [14], but these provide only a generalized shape of the anterior surface, without a detailed investigation of the surface's hills and valleys. We are unaware of any other study that has analysed the three-dimensional geometry of the anterior surface of the patella and its relationship to the cutting plane.

The purpose of this study was therefore (a) to determine the desired resection plane by having surgeons draw virtual resection lines on axial and sagittal preoperative X-rays, (b) to determine the corresponding estimated anterior surface plane, and (c) to determine a contact configuration on the anterior patellar surface that can achieve the desired patellar resection plane.

## 2. Materials and Methods

There were four stages to this study: preparing the images, obtaining surgeon input, analysing the surgeon data, and evaluating different contact geometries to achieve the desired resection plane.

### 2.1. Preparing the Images

Following institutional review board approval, we imaged 18 cadaveric knee specimens (9 left, 9 right; 8 male, 10 female; average age 76; range, 34–91 years) using computed tomography (CT), with a slice thickness of 0.6 mm. All of the specimens had largely normal anatomy, without obvious osteophytes, although some showed signs of early osteoarthritis.

**Figure 2:**
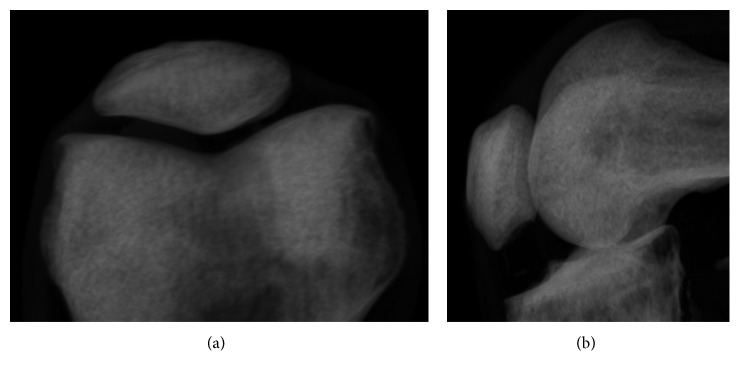
Pseudo-X-rays of a left knee created from a CT scan: (a) axial (skyline) and (b) sagittal (lateral).

Pseudoradiographs were created from the CT scans in the axial and sagittal directions, using Amira image analysis software (version 5.3.1, Visage Imaging, Andover, MA). The CT was viewed using the volume rendering (Volren) function, which was adjusted so that the bone appeared white and the background black. The threshold was set to remove most of the soft tissue in the view to look as similar as possible to a clinical X-ray. The image was rotated to align the knee similar to how a radiation technologist would align a patient: for the axial view, the image was aligned to provide a skyline view, where the patella is viewed from the superior looking downward (Figure 2(a)) (in patients, this requires flexing the knee to be able to view the patella from the top; in creating the pseudo-X-ray, the viewpoint was rotated about the patella until the patellar ridge was aligned with the *z*-axis, i.e., coming directly in/out of the image) (Figure 2(a)); for the sagittal view, the image was rotated until the femoral condyles were aligned, i.e., overlapping, as they would in a typical sagittal X-ray (Figure 2(b)). Screenshots of each view were taken and the orientation saved for later analysis. Three repetitions of each of the projections were saved in randomized order, for a total of 54 axial projections and 54 sagittal projections.

### 2.2. Obtaining Surgeon Input

Four experienced, fellowship-trained orthopaedic surgeons assisted in determining the desired resection plane and the corresponding anterior surface plane. Using a custom macro in ImageJ (version 1.42q, National Institutes of Health (NIH), USA), the surgeons marked the lines electronically on each X-ray; previous lines did not appear. The (*x*, *y*) coordinates of the endpoints of the line were recorded in a text file along with the surgeon's initials and date.

X-ray input was performed in four passes. In the first pass, the surgeon went through the axial projections, drawing the line at which they would ideally resect the patella. In the second pass, they drew a line to represent their best estimate of the anterior surface. Third, they went through the sagittal projections, drawing their ideal resection line, and fourth, they went through the sagittal projections to estimate the anterior surface. All of this was done without concern for thickness: while patellar thickness is an important aspect of the resection, it is set independently by the surgeon during surgery, after aligning to the anterior surface, which is the focus of this study. Mediolateral positioning of the patellar component on the flat cut surface, while also an important aspect of patellar resurfacing (for which slight medialization is recommended [15]), is a separate surgical consideration from the resection plane itself and was therefore not included in this study. Altogether 864 lines were drawn: 4 surgeons × 18 patellae × 2 views × 2 lines per view × 3 repetitions.

### 2.3. Analysing the Surgeon Data

The first step in analysing the data was to understand them visually, which was done by displaying the drawn lines on circumferences of the patellae.

For the quantitative analysis, line angles were measured from the horizontal (mediolateral, ML) axis in the axial views and from the vertical (superoinferior, SI) axis in the sagittal views. For plotting purposes, an average of all the surgeon angles was computed for each patella and this result subtracted from each of the measured angles so that all of the angles were centred about zero. PASW Statistics software (version 17.0, Statistical Package for Social Sciences (SPSS) Inc., Chicago, IL) was used to calculate the interobserver and intraobserver repeatability.

### 2.4. Evaluating Contact Geometries

To establish a 3D plane from the two sets of 2D data (axial and sagittal), the patellae were segmented from the CT scans using Amira. The surface model was then exported as a dxf file to AutoCAD (version 2010, AutoDesk, San Rafael, CA) to create planes along the surgeon-identified resection lines. The alignment of the model was crucial to transferring the surgeon lines accurately to the virtual 3D model; by saving the alignment of the CT earlier, when the 2D projections were created, the 3D model created from the CT was already aligned.

For each patella and each surgeon, a plane was created based on the average of the three repetitions. This was done by drawing a line along the axial view, a line along the sagittal view, and creating a plane along the two lines.

The resection planes, created from the surgeons' input, were visually compared to each other as well as the patellar surface. Without changing alignment, the planes were moved anteroposteriorly (representing different resection thicknesses) to visually inspect how they looked on the models and if there were any landmarks to which they specifically aligned. This visual inspection, along with the surgeons' comments about desiring a symmetric resection, was used to identify a contact configuration to align to the surgeons' desired resection plane.

A variety of contact configurations were tested and compared to the resection planes by modelling the geometry of the contact plane in comparison to the anterior surface and resection planes. Possible contact configurations included different distances and layouts of pegs, a flat plate corresponding to the previously-developed CAS system [11, 12] and a ring corresponding to the existing reamer. Applying flat-jawed callipers, i.e., the standard clinical measurement instrument, to the patella has been shown previously to cause it to rotate [8], showing that this is not a suitable contact mechanism. For each configuration tested, the simulated geometry of the contact model was applied to the anterior surface of the 18 patella models, creating a plane that was compared visually to the surgeon input in the ML and SI directions. Most configurations could be discarded based on a visual comparison between the proposed plane and the desired plane. To quantify the discrepancies of the final selected configuration, the axial resection (AR), sagittal resection (SR), axial surface (AS), and sagittal surface (SS) results were each sorted into three categories: (1) patellae for which the contact-based plane was within the range of surgeon averages for that specimen, (2) patellae for which this plane was within the overall range of surgeon angles for that specimen, and (3) patellae for which the plane fell outside the range of surgeon input for that specimen.

## 3. Results

### 3.1. Surgeon-Defined Resection Planes and Anterior Surface Planes

**Figure 3:**
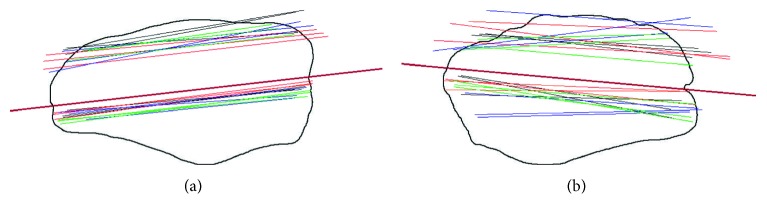
Patellae with the most and least consistently drawn axial resection lines. The four colours represent the four different surgeons, with three repetitions each. The solid black line represents the corresponding peg plane that was later defined (Section 3.2). Thickness is unimportant in the representation since this is decided intraoperatively based on the patellar thickness and component thickness.

**Figure 4:**
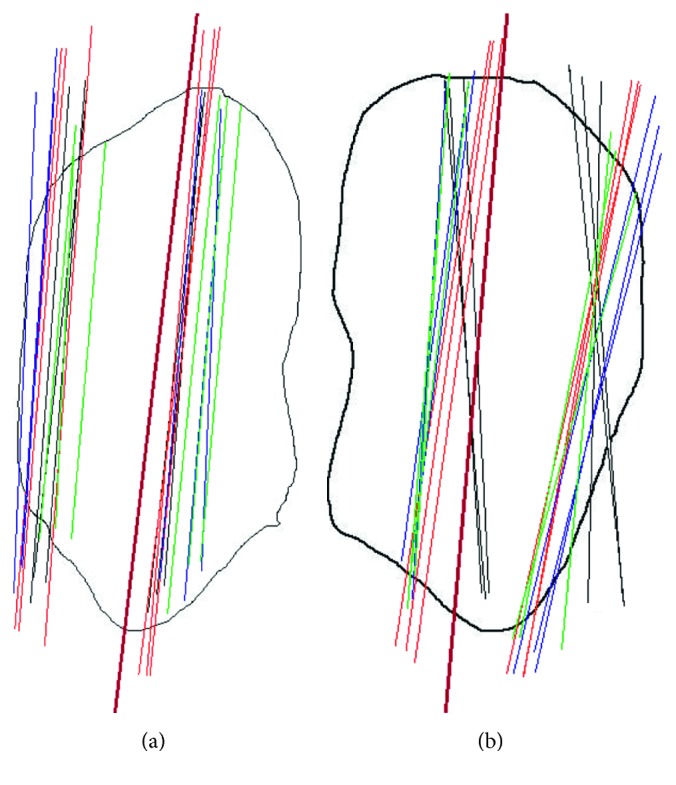
Patellae with the most and least consistently drawn sagittal resection lines. The four colours represent the four different surgeons, with three repetitions each. The solid black line represents the corresponding peg plane defined later (Section 3.2). The thickness is unimportant in the representation since this is decided intraoperatively based on the patellar thickness and component thickness.

There was high variability in the estimated resection lines and anterior surface lines within and between the surgeons. As a visual representation, the most and least consistently drawn lines are shown on the patellar circumferences in Figure 3 (axial) and Figure 4 (sagittal). In general, the flatter the patella, the more consistently the lines were drawn, while the more rounded or irregular the patella, the more inconsistently they were drawn, although this did not hold true in all cases. In general, the more well-defined the medial and lateral extents were on the axial views, the more consistently the lines were drawn, although this also did not hold true in all cases. The surgeons appeared to be taking a mental least-squares fit to the anterior surface but varied in which part of the anterior surface they included.

**Figure 5:**
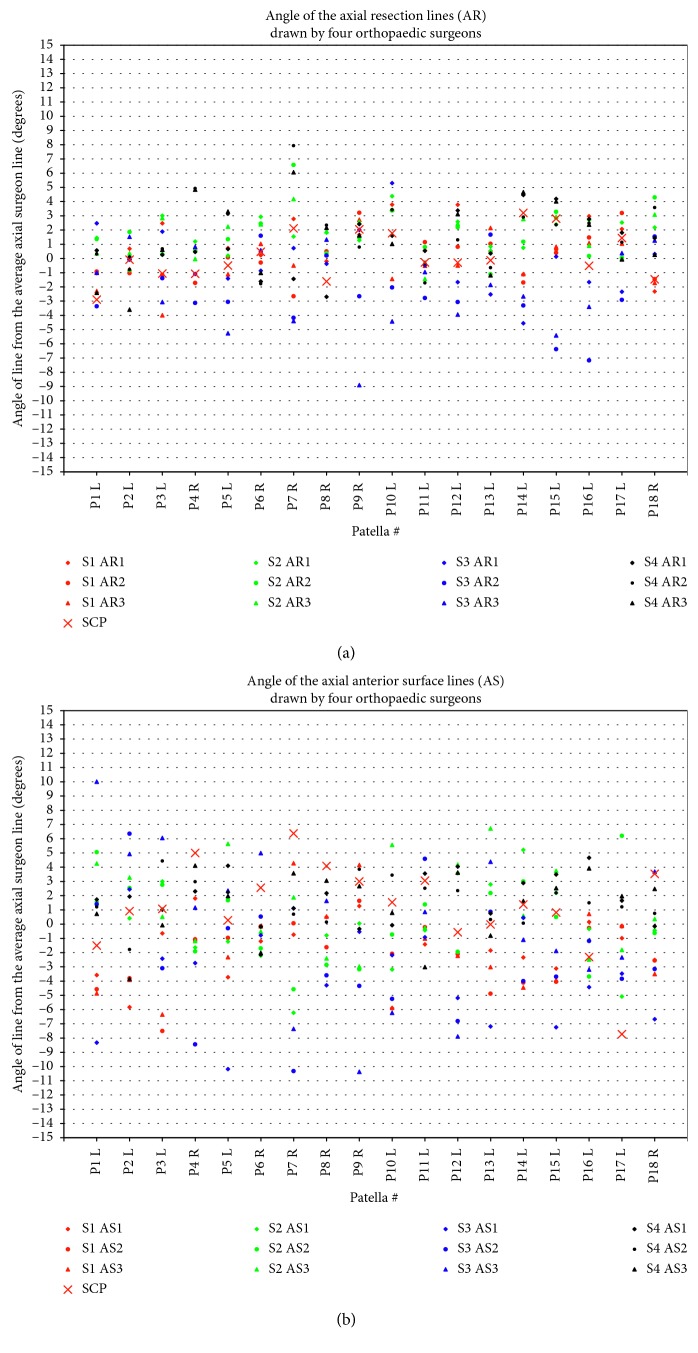
Angle of drawn lines on axial projections: (a) axial resection (AR) lines and (b) axial anterior surface (AS) lines. Positive angles mean the patella is thicker medially. Results are centred around zero for each patella. SCP = symmetric contact points, i.e., the calculated plane based on the final selected configuration (Section 3.2), which is symmetric about the patellar centre. While the SCP planes did not always match the anterior surfaces drawn (b), they were within a few degrees of the desired resection plane (a), which is the primary clinical goal. S1–S4 = surgeons 1–4.

**Figure 6:**
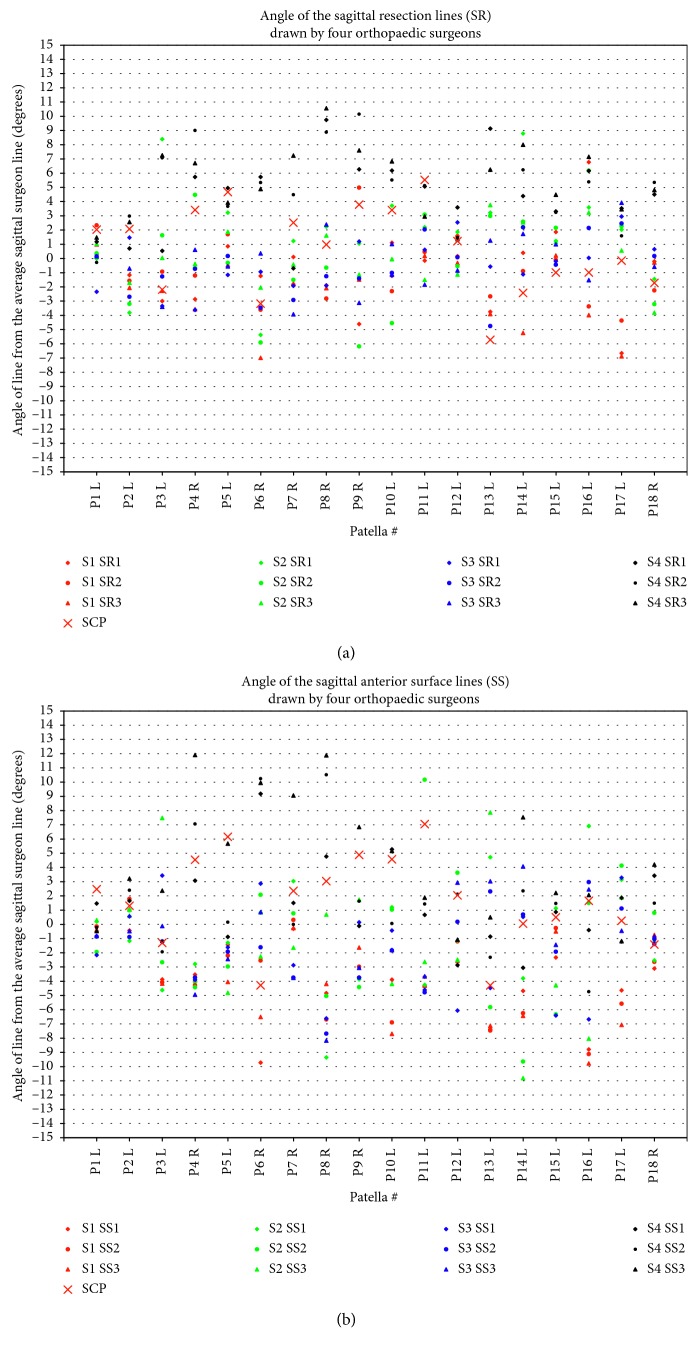
Angle of lines drawn on sagittal projections: (a) sagittal resection (SR) lines and (b) sagittal anterior surface (SS) lines. Positive angles mean the patella is thicker superiorly. Results are centred around zero, i.e., the average, for each patella. SCP = symmetric contact points, i.e., the calculated plane based on the final selected configuration (Section 3.2), which is symmetric about the patellar centre. S1–S4 = surgeons 1–4.

Intrasurgeon repeatability, as judged by the standard deviation, was best for Surgeon 1 (SD = 1.3°), followed by Surgeon 4 (SD = 1.7°), Surgeon 2 (SD = 2.2°), and Surgeon 3 (SD = 2.4°) (Figures 5 and 6). Intersurgeon repeatability was best for the *axial resection line* (SD = 1.5°), followed by the *sagittal resection line* (SD = 1.7°), *axial anterior surface line* (SD = 2.2°), and *sagittal anterior surface line* (SD = 2.2°) (Figures 4 and 5). Perceptions of the resection lines varied by as much as ±9°, and perceptions of the anterior surface varied by as much as ±11°.

The mean angular difference between the *axial resection line* and the *anterior surface* line was 2.4° (SD = 2.1°). The mean angular difference between the *sagittal resection line* and the *anterior surface* line was 3.5° (SD = 3.3°). From the visual display of the lines on the patellar circumferences (see Figures 2 and 3 for examples), it appeared that, when asked to identify the anterior surface, the surgeons focused on a more localized portion of the anterior surface whereas the estimated resection plane approximated a symmetric resection; if the resection plane were to align with the drawn anterior surface plane, it would incorrectly result in thicker medial and superior sides.

### 3.2. Anterior Surface Contact Configuration Selection

The first decision in the process of defining the desired configuration was to use a system of three contact points since three points always find a stable orientation, as with a three-legged stool. With four or more points, a ring, or a flat plate, there is a risk of instability, as with a chair with one shorter leg. In general, the patellar geometry was higher in the middle and sloped off inferiorly, which explains why a ring of contacts (as with the reamer) or a flat plate can create a tilt of several degrees on some patellae [8, 12, 13]. Although early investigations revealed that the flattest portion of the anterior surface was consistently in the superolateral quadrant, the pegs were too close together in this region to provide good stability; furthermore, after analysis, it was discovered that focusing the pegs in this region did not provide a symmetric resection.

The final configuration that fit best with the surgeon-defined resection planes was a 16 mm equilateral triangle, with two points superior (medial and lateral) and one point inferior, centred on the patellar centre. For the cut to be symmetric, the device needed to be central on the patella, and due to the geometry where the inferior edge begins to slope off and narrow, 2 pegs fit best superiorly, with the 3^rd^ peg inferior. The average patella is 46 mm in the mediolateral direction [16] and symmetry is measured 15 mm from the patellar extents [2], which leaves 16 mm in the centre of the patella; thus a 16 mm spacing about the centre was used. This resulted in a good estimate of the desired resection plane.

**Figure 7:**
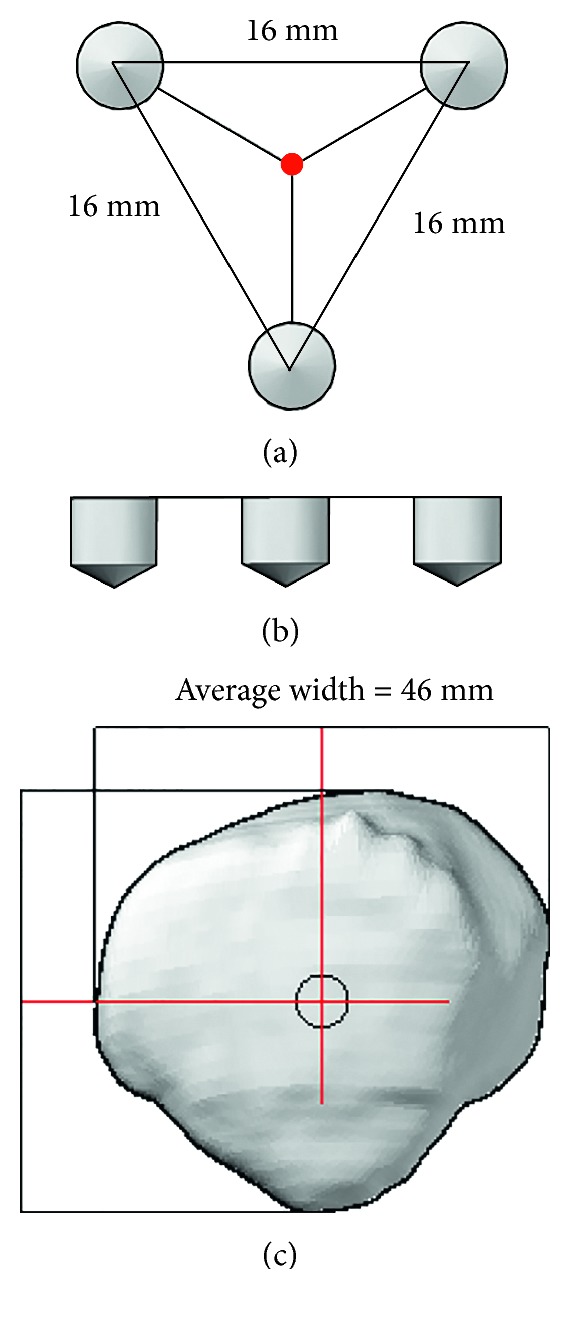
Final peg configuration proposed to align the resection device to the desired resection plane: (a) shows the top view of peg configuration, which is aligned (red dot) to the centre of the patella; (b) shows the side view of the equal-length pegs; and (c) shows the patella view, whereby the patellar centre is defined midway between the mediolateral and superoinferior extents. Patellar width averages 46 mm [16].

The patellar centre was defined halfway between the superior and inferior extents and medial and lateral extents (Figure 7). Having symmetry about the centrepoint further simplifies the design of a surgical instrument. We considered a 15 mm triangle for smaller patellae, but this altered the resulting angles by at most 0.3°, which was too small to be worth the practical consequences. With 16 mm sides, the distance between the inferior and superior points is 13.9 mm.

Using this peg configuration, most of the resection plane and anterior surface plane angles (53 out of 72; 15 AR, 11 SR, 14 AS, and 13 SS) fell into the first category, within the surgeon averages, as expected and desired (Figures 4 and 5); 15 lines (3 AR, 4 SR, 4 AS, and 4 SS) fell into the second category, within the surgeon ranges; and 4 (3 SR, 1 SS) fell into the third category, outside of this range, but only by 1.0°, 0.5°, 0.4°, and 0.6°, respectively.

## 4. Discussion

This study analysed the relationship between the anterior surface of the patella and the surgeons' desired resection planes. On the basis of this shape analysis study, a novel peg configuration was generated for a patellar resection device that was within 1° of the range of angles that the surgeons defined as their desired resection line for all 18 patellae studied, with most falling within the range of surgeon averages. Subsequent use of this configuration in a device designed to detect patellar asymmetry intraoperatively resulted in good accuracy in comparison to CT scans [17]. Note that if later clinical evidence supports patellar resection at an angle other than parallel to the anterior surface, extending one or two of the pegs can achieve this angle easily.

The high variability in surgeon input confirms the difficulty of defining the anterior surface plane and of defining an ideal resection plane. It also highlights the difficulty of reporting patellar asymmetry or patellar tilt clinically since the definition of the patellar horizon is so variable, despite the fact that studies describe it as a fixed reference. Conversely, the high variability provides some leeway in achieving an exact resection.

The intrasurgeon and intersurgeon repeatability were similar to a previous study with three different surgeons, in which the AR intrasurgeon repeatability using the medial-lateral extents method was 1.6°, and the AR intersurgeon repeatability was 2.0°, although the maximum differences were larger in the present study [8]. The present study is novel in evaluating sagittal radiographs and the anterior surface, as well as in investigating the relationship between the 3D anterior surface geometry and the desired resection plane. Sagittal symmetry is important because SI asymmetry has an even stronger correlation with anterior knee pain than ML asymmetry [2]. The same 2D-3D analysis techniques could be used for any joint in which surgeons routinely use plain X-rays.

The only quantitative definition of asymmetry that we are aware of is a difference of greater than 2 mm between the medial and lateral or superior and inferior patellar thicknesses, measured 15 mm in from the patellar extents [2, 3]. On an average sized patella [16], this results in a similar ML spacing to our peg configuration, although as previously mentioned, a 3-peg configuration was chosen since it is more stable and predictable than a 4-peg configuration.

The main limitation of this study is that the surgeons' lines drawn on X-rays may not be the same plane as they would choose to resect intraoperatively. Nevertheless, it reflects what they would like to see on the X-ray postoperatively and is the basis for measuring asymmetry. Only one of the surgeons (Surgeon 1) routinely does preoperative planning for the patella (axial only) and was therefore most familiar with drawing lines on the X-rays; he had the smallest standard deviation and his resection lines tended to be closer to the overall surgeon average. The particular projection of the patella could also impact the resulting plane; however, all surgeons saw the same projections, so the variability within and between surgeons is unrelated to the projection. Another potential limitation is that the patellae investigated were largely healthy patellae, but since arthritis affects the posterior surface rather than the anterior surface, this should not represent a problem: a benefit of the device is that the peg configuration should produce the desired resection on a diseased patella equally well to a healthy one. The shape of the articulating patellar surface and whether it is a male or female patella, which have previously been shown to affect resection accuracy clinically [8], should likewise have no impact on the result since the device references solely off of the anterior surface.

## 5. Conclusions

Based on a rigorous and extensive shape analysis of the patella, a peg configuration was derived to achieve the surgeons' desired resection plane relative to specific contact points on the anterior surface. By analysing numerous alternative configurations on the 18 patellae, the best peg configuration consisted of a 16 mm equilateral triangle centred on the patella, which has been adopted into and successfully tested on two prototype surgical devices [17, 18]. The resulting configuration could also benefit custom rapid-prototyped surgical guides (which have often been used for the femur and tibia), by working from similar contact points. Furthermore, existing surgical devices could be modified to incorporate the proposed contact configuration.

Improving patellar symmetry will help reduce the number of patients who experience postoperative pain and reduced function. Using the proposed peg configuration in a patellar resection device should improve accuracy while remaining simple, fast, and noninvasive to use.

## Data Availability

The data used to support the findings of this study are included within the article.
